# Cerasomes and Bicelles: Hybrid Bilayered Nanostructures With Silica-Like Surface in Cancer Theranostics

**DOI:** 10.3389/fchem.2018.00127

**Published:** 2018-04-18

**Authors:** Sadaf Hameed, Pravin Bhattarai, Zhifei Dai

**Affiliations:** Department of Biomedical Engineering, College of Engineering, Peking University, Beijing, China

**Keywords:** hybrid materials, nanotechnology, cerasomes, bicelles, cancer theranostics

## Abstract

Over years, theranostic nanoplatforms have provided a new avenue for the diagnosis and treatment of various cancer types. To this end, a myriad of nanocarriers such as polymeric micelles, liposomes, and inorganic nanoparticles (NPs) with distinct physiochemical and biological properties are routinely investigated for preclinical and clinical studies. So far, liposomes have received great attention for various biomedical applications, however, it still suffers from insufficient morphological stability. On the other hand, inorganic NPs depicting excellent therapeutic ability have failed to address biocompatibility issues. This has raised a serious concern about the clinical approval of multifunctional organic or inorganic-based theranostic agents. Recently, partially silica coated nanohybrids such as cerasomes and bicelles demonstrating both diagnostic and therapeutic ability in a single system, have drawn profound attention as a fascinating novel drug delivery system. Compared with traditional liposomal or inorganic-based nanoformulations, this new and highly stable nanocarriers integrates the functional attributes of biomimetic liposomes and silica NPs, therefore, synergize strengths and functions, or even surpass weaknesses of individual components. This review at its best enlightens the emerging concept of such partially silica coated nanohybrids, fabrication strategies, and theranostic opportunities to combat cancer and related diseases.

## Introduction

Nanotechnology-based therapeutic approaches have received immense attention in the treatment of cancer and several other pathological disorders (Bhattarai et al., 2018). A plethora of nanocarriers with uniquely appealing physiochemical features, specific functionalities and applications have been engineered for the diagnostic and therapeutic treatment of cancer and related diseases (Alexis et al., 2008; Dhar et al., 2008; Hillaireau and Couvreur, 2009; Parveen et al., 2012; Bhattarai and Dai, 2017; Gao et al., 2017a,b). At present, more than 51-conventional therapeutic nanocarriers such as liposomes, polymeric micelles and albumin NPs are already approved by FDA while many of them are in the final stage of their clinical approval process for the treatment of devastating cancer (Cho et al., 2008; López-Dávila et al., 2012; Bobo et al., 2016). However, despite rapid progress in the design and application of novel therapeutic nanoagents, controlled drug delivery via these nanocarriers in a complex biological system is still a major challenge. Moreover, several other critical issues, including morphological instability in blood circulation, insufficient drug loading, and off-target drug release further limit their therapeutic efficacy *in vivo* (Kamaly et al., 2012; Jin et al., 2016).

To resolve this problem, a new and different class of hybrid structure comprising of organic and inorganic counterparts have emerged as a valuable tool for several intriguing biomedical applications (Liang et al., 2015). Such hybrid nanoplatform not only combine the intrinsic properties of organic and inorganic building blocks but also potentially attain new chemical, physical and biological function through supramolecular interactions among them, thus providing safer and efficient treatment options than conventional nanocarriers (Benyettou et al., 2011; Jin et al., 2013). In general, these multifunctional nanocarriers have potential to improve the therapeutic index by targeting multiple pathways, minimizing systematic toxicity by reducing the leakage of the chemotherapeutic drug in blood circulation, and delivering the chemotherapeutic drugs specifically to the tumor (Jin et al., 2012). More recent evidence in the progress of hybrid nanocarriers encompasses the integration of imaging capability (either attached on the surface of these hybrids or incorporated into their internal domains) for either pre-operative or post-operative diagnosis of tumor, or facilitate pharmacokinetic and pharmacodynamics studies as a part of drug delivery applications responsible to minimize drug cytotoxicity in normal tissues (Prakash et al., 2011; Hu et al., 2013; Luo et al., 2014; Song E. et al., 2014; Zeng et al., 2014; Wang et al., 2016). In brief, these hybrid nanocarriers hold tremendous potential to overcome the limitation of traditional chemotherapy (e.g., rapid clearance, high toxicity, and non-specific cell/tissue distribution; Wakaskar, 2018).

**Figure 1:**
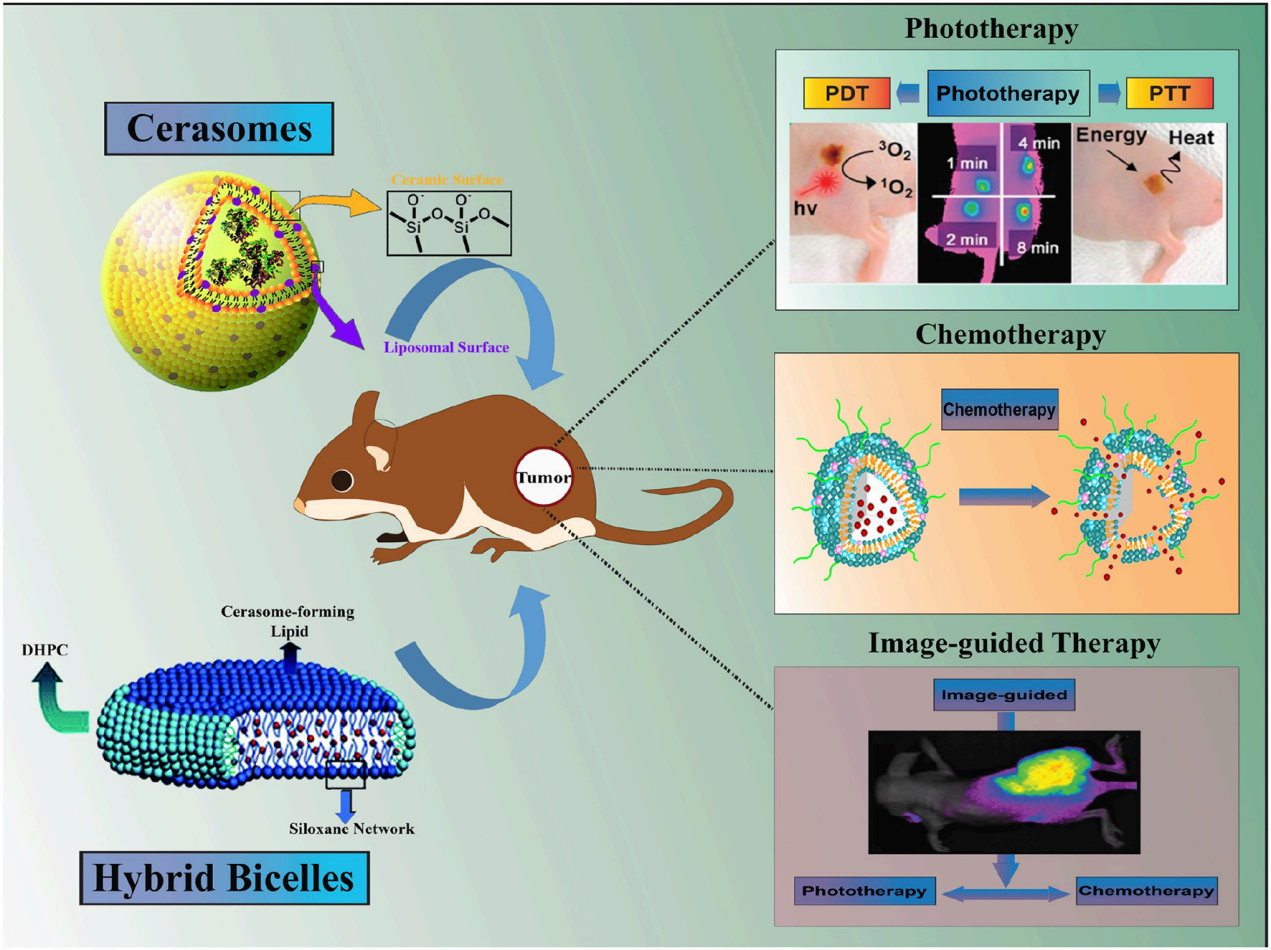
Schematic illustration of Hybrid Bilayered Nanostructures with Silica-like Surface in Cancer Theranostics.

Among these nanohybrid platforms, liposomes like partially silica coated bilayered nanohybrids (∂SiNHs), namely cerasomes and bicelles, have witnessed a considerable rise in the broad spectrum biological applications such as drug delivery, diagnosis, and treatment of cancer and related diseases (Yasuhara et al., 2012; Jin et al., 2014). Such ∂SiNHs nanohybrids are of immense interest owing to the advantages such as low toxicity, controllable size and shape, biocompatible, and good stability in a physiological condition (Jin et al., 2012). Additionally, dense siloxane network on the surface of ∂SiNHs are capable of rendering a multifunctional ability such as loading of a wide variety of therapeutic molecules, imaging moieties and/or surface functionalization via targeting ligands, in a most convenient and facile way (Du et al., 2018; Figure 1). This review at its best summarizes recent progress in the design, development, and application of multifunctional cerasomes and bicelles, particularly in cancer theranostics. Moreover, the advantages of nanohybrid carriers and challenges impeding their progress in cancer theranostics highlighted in this review could benefit the broad scientific community to stimulate further research in this area.

## Nanocarriers: recent advances and challenges

### Conventional nanocarriers

In the race to combat cancer, a great deal of effort has been made in the fabrication of highly biodegradable and biocompatible organic nanocarriers (López-Dávila et al., 2012). Majority of these organic nanocarriers are synthesized from conventional bio-precursors such as lipids and polymers (Peer et al., 2007). Polysaccharides, derived from the monosaccharides, that are connected by a glycosidic bond is a representative example of this class. They are naturally occurring polymers in animals (e.g., chondroitin sulfate and chitosan), plants (e.g., Guar gum and Pectin), and microorganisms (e.g., Dextran) (Chayed and Winnik, 2007). The hydrolysis of glycosidic bond facilitates the release of the drugs from polysaccharides based NPs at the targeted sites. Chitosan, a copolymer of glucosamine and N-acetylated glucosamine, has emerged as the most valued polysaccharides in the fabrication of NPs for drug delivery and related applications. Recently, chitosan-based organic-inorganic nanohybrid systems with favorable biological properties such as biocompatibility, biodegradability, and non-toxicity have been investigated for stimuli-responsive drug delivery application (Prakash et al., 2011; Popat et al., 2012; Ribeiro et al., 2014). To date, lipid-based carriers particularly liposomes have been extensively investigated as the most promising nanocarrier domain (Paliwal et al., 2015). Liposomes are versatile nanocarriers for loading hydrophobic and hydrophilic drugs with better pharmacokinetics (Torchilin, 2014). Additionally, since the liposomal nanocarriers are composed of phospholipids, they possess inherent ability to degrade easily in a biological system (Pattni et al., 2015). However, major drawbacks associated with the liposomal nanocarriers is their physiological instability resulting in a noticeable drug leakage *in vivo* owing to stringent rheological properties that weaken the molecular interactions between the phospholipid components (Blanco et al., 2015). To overcome this limitation, an attempt was made to functionalize the surface of liposomes with hydrophilic poly(ethylene glycol) chains (PEGylation). Although PEGylation furnished the liposomes with long circulating properties, but manifest some unwanted attributes like skin toxicity.

Other prominent conventional nanocarriers that shared the spotlight with lipid-based nanocarrier are polymeric, mesoporous silica, and different metals, i.e., iron, silver and gold nanocarriers (Tang et al., 2012; Ulbrich et al., 2016). Smaller particle size, improved drug loading, structural integrity and controlled release of therapeutics offers a huge advantage for cancer therapeutics. However, these nanocarriers have some limitations in term of toxicity, and poor drug entrapment resulting in maximum drug leakage before reaching to the targeted site (Kreuter, 2014; Masood, 2016). Nevertheless, these NPs still hold top priorities in the design of novel nanoformulations and are explored widely.

### Hybrid nanocarriers

The increasing interest for robust nanocarriers with novel functionalities has motivated researchers for the inception of hybrid nanoplatforms that utilizes the positive attributes of organic and inorganic components (Vallet-Regí et al., 2011). The organic counterpart complements higher drug loading, tumor-specific disintegration and the delivery of cargos in deep-suited tumors for both imaging and therapy (Liu et al., 2015; Li et al., 2018). On the other hand, inorganic components endow the nanohybrid particles with additional stability, enhanced blood circulation time and tumor targeting abilities (He et al., 2015). In a representative work, Chen et al. designed a self-assembled Fe_3_O_4_ core/polymer shell hybrid nanostructure loaded with a chemotherapeutic agent, DOX (Chen et al., 2014). The integration of pH-responsive moieties, such as an acid-labile bond in the nanohybrid, allowed the pH-dependent release of DOX, particularly in the acidic tumor environment. In another work, Wu et al. fabricated a liposomes@SiO_2_@Au nanohybrid for the delivery of DOX (Wu et al., 2011). This hybrid nanoplatform facilitated higher loading of DOX, in addition, deployed NIR laser to excite the gold nanoshell for the activation and release of DOX via local heating *in vivo*. Similar nanohybrids combining stimuli-responsive inorganic NPs and non-thermo-sensitive organic components, for e.g., polymeric micelles encapsulated with UCNPs, have also been fabricated to trigger the release remotely (Yan et al., 2011). The selection of different organic/inorganic components to self-assemble within the nanohybrid is the most crucial step and is usually dependent on envisioned therapeutic goal, type of drugs, imaging agents, expected route of administration and biocompatibility of individual components (Wang et al., 2018). As an emerging platform, partially silica-coated organic-inorganic nanohybrid (cerasomes) exhibit integrated properties of traditional lipid-based NPs and inorganic silica NPs. Cerasomes are biomimetic colloidal NPs with an additional layer of the polyorganosilxane network which imparts cerasomes with higher stability than conventional liposomes (Jin et al., 2014). Nano-sized cerasomal NPs have various advantages, including biocompatibility, prolonged blood circulation, higher tumor accumulation by enhanced permeability and retention (EPR) effect, and decreased reticuloendothelial system (RES) clearance (Jin et al., 2014). Thus, cerasomal NPs are capable of overcoming pharmacological limitations with substantial advantages over conventional nanocarriers (Yue and Dai, 2014). The following section will highlight the emergence of a new type of nanocarriers such as cerasomes/bicelles that can overcome the major challenges of conventional nanocarriers.

## The emergence of partially-silica coated hybrid nanocarriers: cerasomes

### Design and synthesis of cerasome-forming lipid and cerasomes

The fabrication technique of cerasomes with multilamellar vesicle structure is conceptually analogous to that of phospholipid-based liposomes. In general, cerasome-forming lipids (CFLs) are composed of three basic components: hydrophobic alkyl chains, hydrophilic lipid molecules with triethoxysilane headgroups, and a connector unit, which is particularly important for establishing intermolecular hydrogen bonding between the hydrophilic and hydrophobic part to impart additional physiological stability to cerasomes. Considering these point, Kikuchi et al. designed a series of CFL through a simple condensation reaction using three molecular units, dihexadecyamine, succinic anhydride, and 3-aminopropyltriethoxylsilane (Kiyofumi et al., 1999; Katagiri et al., 2007). Furthermore, Kiyofumi et al. successfully designed and fabricated organic-inorganic nanohybrid cerasomes based on organoalkoxysilane with lipid-like architecture via sol-gel reaction and self-assembling methodology (Kiyofumi et al., 1999). Recently, numerous morphologically stable cerasomal NPs have already been fabricated by employing inexpensive synthetic approaches such as ultrasonic dispersion, ethanol-sol injection and thin film hydration method (Liang et al., 2013). A common synthesis strategy such as thin film hydration involves dispersion of the liquid in a solution comprising mostly of a thin film which would later take shape of spherical vesicles with an aqueous center. Specifically, thin film method involves the dissolution of the CFL in an organic solvent, evaporation of the solvent, and the dispersion of the obtained CFL film in an aqueous media (Jin et al., 2012). Similarly, ultrasonic dispersion method follows the direct dispersion of CFL in an aqueous medium followed by vortex mixing and ultrasonication. Alternatively, the ethanol-sol injection method involves the hydrolysis of CFL by incubating in an acidic ethanol solution for a specific time. The sol thus obtained is injected into water to yield cerasomal NPs (Katagiri et al., 2003). In the past few years, our group have consecutively synthesized and evaluated series of a new class of CFL by altering the number of triethoxysilane headgroups and hydrophobic alkyl chains in the formulation (Ma et al., 2011). The detailed synthesis approach, morphological characterization and biological application of these new CFL based cerasomes have already been summarized in our previously published review article (Yue and Dai, 2014).

### Functional properties of cerasomal NPs

The unique physiological and biological properties of cerasomes have accredited them as a fascinating platform for cancer theranostics (Jin et al., 2014; Ding et al., 2015; Fu et al., 2015). The sol-gel processed cerasomes with precise nanostructure are inevitable choice in cancer therapy mainly due to: (a) the possibility to incorporate various hydrophilic/ hydrophobic payloads with higher encapsulation efficiency and loading capacity, (b) the ease of tuning their internal chemistry (composition) and surface properties can allow further conjugation of imaging moieties, and (c) the excellent cellular and tissue biocompatibility of cerasomes can attenuate the toxic effect related to the silica NPs. In addition to this, the rationale for the use of cerasomes as an extraordinary theranostic platform mainly encompasses other three major aspects: (i) high remarkable stability, (ii) modifiable surface chemistry, and (iii) biocompatibility and biodegradability that needs further discussion (Liang et al., 2013).

#### Size control

The size of nanocarriers is often implicated as a key parameter that determines the systematic distribution, cellular internalization, tumor penetration and therapeutic efficacy of NPs (Salatin et al., 2015). Nanoparticles of sizes <200 nm are only permissible in the tumor regions depicting EPR effect. Therefore, design and fabrication of nanocarriers that is expected to have application in cancer theranostics should meet this size-requirement and should be dealt with great attention (Ding et al., 2015; Kumari et al., 2016; Ojha et al., 2017). In our preliminary finding, cerasomal NPs intrinsic constituents such as triethoxysilane headgroups or alkyl chains attributed for vesicular architecture were identified as a major role player in manipulating the size and other important parameters such as encapsulation efficiency, drug loading capacity and release of payloads (Liang et al., 2013). Cerasomes of mean hydrodynamic diameters (D_hy_) 140–230 nm were synthesized by a simple sol-gel reaction and self-assembly process. In particular, cerasomes without loading any drugs (referred as blank) showed relatively smaller diameter NPs with the increasing number of triethoxysilane groups. This can be mainly attributed to the higher degree of polymerization of the siloxane resulting in compact cerasomal NPs that can be easily achieved by altering internal constituents of cerasome forming lipids. In contrast, size of cerasomal NPs can also be controlled extrinsically by simply changing the ratio of phospholipids such as DSPCs that are responsible for preparation of cerasomal NPs, similar to the conventional liposomes. Taking account of these parameters, we synthesized a nanohybrid cerasomes for the delivery of paclitaxel (PTX) to the tumor site by simply adjusting the vesicle composition (Cao et al., 2012). In this study, the addition of DSPC revealed size-dependent properties of cerasomal NPs. In general, cerasomes displayed an average D_hy_ of 190.3 ± 10.5 nm in the absence of any DSPCs, however, the addition of only 10% DSPC in the composite vesicles induced an observable change in the size of cerasomal vesicles. In contrast, a further increase in the content of DSPC resulted in the formation of cerasomal composites (CCs) that were significantly larger than cerasomes without having any DPSCs, for instance, cerasomes containing 50% CFL and 50% DSPC exhibited D_hy_ of 239.2 ± 28.2 nm. This change in particle size was attributed to the possible alteration in the packaging and polymerization of CFL and susceptible to change with the alteration of the lipid constituents. In summary, it is quite evident that cerasomes can be easily designed and synthesized with optimal sizes by just changing the reactant ratios and are conducive for the use as any other conventional nanocarriers in wide ranges of applications.

#### Surface modification

**Figure 2:**
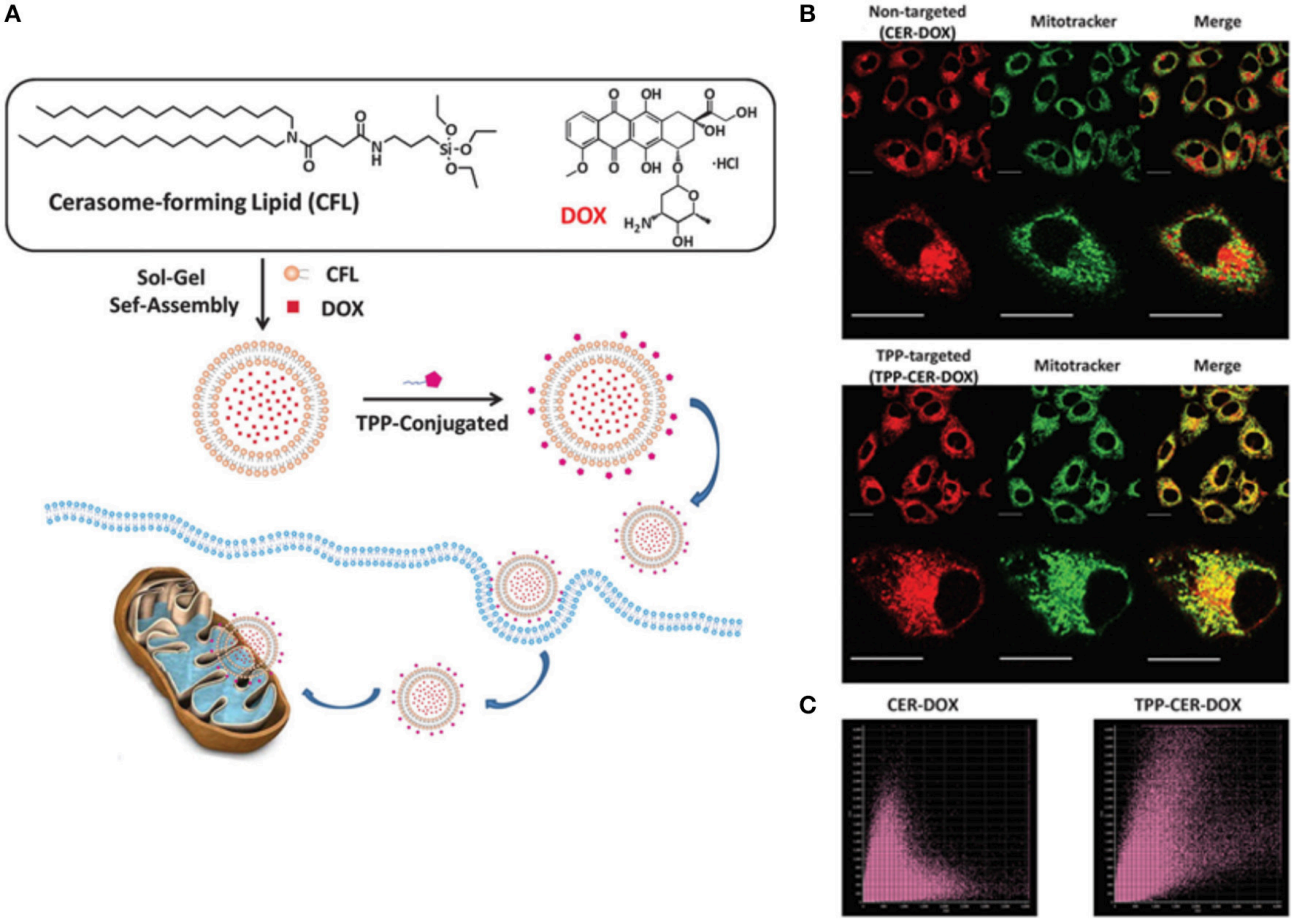
**(A)** Schematic illustration of the formation process of the doxorubicin-loaded cerasomes (CER–DOX) and TPP modified cerasomes (TPP–CER– DOX). Mitochondrial localization of CER–DOX and TPP–CER–DOX. HeLa cells were exposed to non-targeted CER–DOX and TPP-targeted TPP– CER–DOX for 4 h (scale bar: 20 mm). The cells were then stained with mitochondrial marker Mito-Tracker Deep Red for 30 min. The staining solution was washed with pH 7.4 PBS and the cells were observed by fluorescence microscopy. **(B)** The merge images showed that the overlap of Mito-Tracker (green) and CER–DOX (red) was unobvious (top), while the overlap of TPP–CER–DOX was significant (down). **(C)** The Pearson's coefficient analysis plots of CER–DOX (left) and TPP–CER–DOX (right) (Wang et al., 2015).

Nanoparticles differing in the surface properties are likely to have different *in vivo* properties for e.g., pegylated vs. non-pegylated, metallic NPs with a polymeric coating vs. without coating, have different blood half-life time and cellular uptake or renal clearance properties. In this regard, cerasomes facile design strategies offer a unique advantage to modify their surfaces resulting in better interactions with the biological systems. In cancer theranostics, surface modification is crucial to impart some unique characteristics to nanocarriers allowing them to sense specific proteins and cell surface molecules at the tumor site. Specifically, due to the presence of siloxane oligomers, the cerasomes surface can be modified either by simple physical adsorption or covalent conjugation, thanks to the silane chemistry (Hashizume et al., 2010). For example, the addition of 3-aminopropyltriethoxysilane (APS) to a native polyanionic cerasomes can induce charge reversal resulting in a polycationic surfaced cerasomes. Likewise, studies have shown that small molecules, proteins, peptides, and antibodies can also be grafted onto the surface of cerasomes for targeted cancer therapy (Sperling and Parak, 2010). With the aid of targeting ligands, cerasomes can bind to a specific cell surface receptor and are easily uptaken via receptor-mediated endocytosis mechanism. As targeting agents, antibodies possess two epitope binding sites in a single molecule, that endow them with high selectivity and avidity to their target receptors (Bazak et al., 2015). For instance, cerasomes modified with antibodies can bind to the cell surface receptors, providing efficient targeted delivery. In a study by our group, a fluorescent-tagged-antibodies immobilized on the surface of cerasomes were found to enhance the targeted delivery of cerasomes (via receptor-mediated endocytosis) with higher selectivity (Stamm et al., 2011). The presence of silanol networks on the surface of cerasomes, as compared to the conventional liposomes, enables the easy immobilization of biomolecules that are specific to tumor cells to selectively deliver therapeutic moieties to cancer cells with enhanced targeting capabilities. In a study, Wang et al. investigated the highly stable triphosphonium (TPP)-surface modified cerasomes for mitochondrial targeted delivery of anti-cancer drug DOX (Wang et al., 2015). The TPP–targeted cerasomes could easily penetrate across the mitochondrial membrane and accumulate in mitochondria matrix due to electrostatic attraction between TPP and mitochondrial membrane. Thus TPP-CER-DOX exhibited more pronounced anticancer effects as compared to the non-targeted cerasomes (Figure 2). In another study, Leung et al. functionalized the surface of immuno-cerasomes by chemically conjugated anti-epidermal growth factor receptor monoclonal antibody (anti-EGFR mAb) to deliver drugs and imaging agents selectively to the tumor cells (Leung et al., 2014). Anti-EGFR conjugated cerasomes demonstrated enhanced internalization in highly EGFR expressed cancer cells thereby resulting in effective inhibition of tumor growth. Recently, Du et al. developed a similar cerasomal system but using an anti-PD-L1 antibody to provide a molecular target (Du et al., 2018). An anti-PD-L1 antibody conjugated to cerasomes exhibited better anti-tumor activity in comparison to non-targeted cerasomes. Hence, surface modification of cerasomes can unleash exceptional tumor targeting ability and thereby greatly minimize the adverse side effect invited by off-targeting of NPs to normal tissues.

#### Stability and biocompatibility considerations

Stability and biocompatibility are probably the most critical parameters in the design of nanocarriers for effective and safe biomedical application. The siloxane coating offers cerasomes with remarkably higher morphological stability toward long-term storage and surfactant solubilization (Jin et al., 2014). Additionally, cerasomes are comparatively more stable than other nanocarriers in the various biological environment. In the biocompatibility context, nanocarriers must depict limited/negligible toxicity to the organism at its effective dose, perform its function without interfering the healthy tissues and have sufficient circulation half-life to accomplish its intended task. Cerasomes possess biocompatibility as good as those of conventional liposomes but better than the silica NPs, indicating their great potential in the therapeutic application (Ma et al., 2011). In the past few years, our group has explored various strategies to evaluate the *in vitro* and *in vivo* biocompatibility of drug-loaded cerasomes (Cao et al., 2010; Liang et al., 2013; Yue and Dai, 2014). Our investigation on the viability and proliferation of various mammalian cells indicated that these properties were not affected by the internalization of cerasomes at concentrations below 0.2 mg/mL. Nevertheless, the cell viability was decreased to 80% when human umbilical vein endothelial cells (HUVECs) was incubated with 0.2 mg/mL of cerasomes for 24 h, indicating that biocompatibility of cerasomes remains to be improved (Ma et al., 2011). Moreover, it is important to mention that application of cerasomal NPs in cancer theranostics have only a very short history, therefore, it is difficult to assert any concrete conclusion regarding its long-term biocompatibility and biodegradation *in vivo*.

### Application of cerasome-based NPs in cancer therapy

#### Cerasomes in drug and gene delivery

A desirable trait of nanoformulation for cancer therapy is their ability to transport the therapeutic agents particularly to the tumor site for an optimal effect. The intrinsic properties of cerasomal NPs such as biocompatibility, biodegradability, high blood stability, and controlled cargo release, make these NPs interesting for therapeutic application, especially in drug and gene delivery. The potential use of cerasomal NPs in drug delivery encompasses either passive or active accumulation to diseased and inflamed tumor cells with great precision.

##### Controlled release of drugs in tumor

Cerasomal NPs, as compared to the traditional liposomes, can encapsulate both hydrophilic and hydrophobic drugs, moreover, both *in vitro* and *in vivo* release profile can be tuned by regulating the degree of condensation and gap of polyorganosiloxane surface. Hence, we demonstrated for the first time that cerasomal NPs have several unique features to be used as a promising drug delivery system. In this context, Cao et al., investigated and compared the controlled release behavior of paclitaxel (PTX) from nanohybrid cerasomal NPs (PLCs) against liposomal NPs (PLLs) (Cao et al., 2010). Interestingly, PLCs could release nearly half (58.2%) of the amount of PTX at the longest time interval of 120 h while PLLs already released almost all (98%) of the drugs. This slower drug release rate for cerasomes was attributed to the dense siloxane networks which limited the flow of the lipid bilayer membrane, resulting in blockage of drug release channels and subsequent decrease in the drug release rate. Nevertheless, unlike several conventional nanocarriers, the exceptional drug tuning ability of cerasomes is praise worthy. The vesicular composition of cerasomal NPs is a key factor to modulate the sustained drug release behavior from the cerasomes. In order to study the correlation between lipid compositions and corresponding drug release rates, Jin et al. prepared a lipid-mixed cerasomes (dipalmitoylphosphatidylglycerol, DPPG) loaded with a chemotherapeutic drug, doxorubicin (DOX) (Jin et al., 2012). In a normal physiological condition, cerasome NPs exhibited a low initial burst (within 5 h) followed by a sustained release of DOX (over 150 h). However, with the gradual increase in the amount of DPPG on the surface of cerasomes greatly affected the release of DOX *in vitro*. Both, the magnitude of initial burst release and sustained release of DOX from cerasomes increased with respect to the increasing content of DPPG in the formulation. This can be mainly due to the enhanced membrane permeability and weakened interaction between DOX and cerasomes containing DPPG. In addition to the small drug molecules, cerasomal NPs can also be used to deliver siRNA to specific tumor cells, to possibly block the production of disease-causing proteins. This can be accomplished by creating positively charge scaffold on the surface of cerasomes, which allows the binding of negatively charged DNA or siRNA through electrostatic interaction. In a recent work on PEGylated cationic cerasomes (PCCs), we have highlighted the potential of cerasomal NPs for safe and efficient siRNA delivery *in vivo*. To this end, a cationic lipid with hydroxyl group was doped into the PEGylated cerasomes to overcome many of limitation associated with current gene delivery platform. Highly dense polyorganosiloxane network on the PCCs surface imparts higher morphological stability than conventional liposomes. The PEGylated PCCs could prevent the cerasomes from agglomeration and macrophage capture consequently prolong the blood circulation half-life and improve the efficacy of siRNA delivery. Moreover, PCCs have elucidated effective delivery to the liver and preferential uptake by hepatocytes in mice, therefore leading to high siRNA gene-silencing activity. This indicates the high potency of PCCs as a delivery platform for therapeutic oligonucleotides (Jin et al., 2014).

##### Stimuli-responsive cerasomes for enhanced drug delivery

Nanocarriers delivered to the tumor regions are basically accumulated by either passive or active strategies. So far, tumor accumulation alone needn't necessarily improve the therapeutic efficiency, instead, the release of cargos at tumor sites is also equally important. In order to achieve better therapeutic efficiency, drug release in the tumor extracellular space or intracellular region can be modulated by the application of extrinsic or intrinsic stimuli (Karimi et al., 2016). Recently, our group has investigated drug release behavior (both spatial and temporal) from cerasomal NPs in the presence of external stimuli such as pH, light, magnetic field, ultrasound (Xiao et al., 2015; Manigandan et al., 2017). In a pioneer work, Liang et al. synthesized a photo-responsive nanohybrid cerasomes containing a cis-azobenzene unit that worked as an on-off valve in response to an optical stimulus (Liang et al., 2011b). Upon exposure to a UV light (λ = 365 nm), the cis-azobenzene moiety inserted in the cerasome bilayer greatly facilitated the release of an encapsulated guest molecule, Nile red, but was completely inhibited in the dark. This was mainly attributed to the photo-isomerization of azobenzene moiety in the presence of specific wavelength of light which in turn increased the permeability of cerasomal bilayer. Though we didn't demonstrate the bioapplicability of this photo-responsive system, we executed a “proof of concept” work for the photo-responsive release of cargoes from the nanohybrid cerasomes, which has opened new opportunities for cancer therapeutic application and many more.

Other than using light stimulus for the release of therapeutic agents from the nanohybrid cerasomes, we also investigated the magnetic field-responsive nanohybrid cerasomes for controlled release of DOX (Cao et al., 2013). The DOX-loaded magnetic cerasomes (DLMNs) were fabricated by incorporating oleic-acid modified Fe_2_O_3_ NPs and DOX into the cerasomes at various Fe_2_O_3_/CFL ratio. *In vitro* cellular uptake study revealed that an external magnetic field (MF) enabled rapid and efficient uptake of DLMNs by the cancer cells, resulting in the higher capability to inhibit cancer cell proliferation as compared to non-magnetic drug loaded cerasomes. At higher concentration (20μM), the cell viability of cancer cells, without an external MF, was evaluated to be 56.49 ± 3.41% and 63.89 ± 3.45% for DLMCs and DLCs respectively. Interestingly, when an external magnetic field was applied, the cell viability of cancer cell decreased by 27.28 ± 6.89% for DLMCs. This study demonstrates an excellent non-invasive application of the nanohybrid cerasomes for cancer therapy.

**Figure 3:**
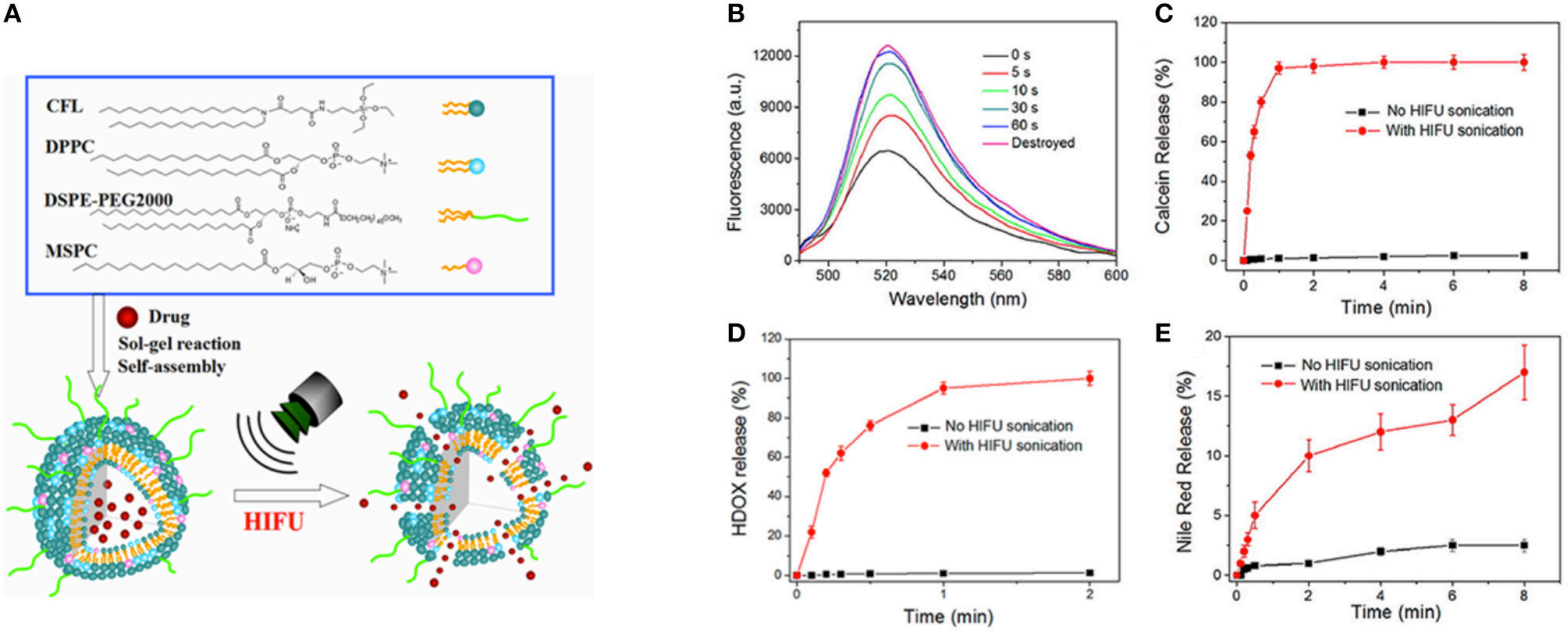
**(A)** Schematic illustration for the formation of drug loaded HTSCs and the drug release from HTSCs upon HIFU sonication. **(B–E)** HIFU triggered drug release profile of HTSCs. **(B)** Fluorescence spectra of the calcein loaded HTSCs-3; **(C)** release profile of the calcein loaded HTSCs-3; **(C)** Release profile of the Nile red loaded HTSCs-3; **(D)** release profile of the HDOX loaded HTSCs-3 with a working voltage of 190 mV and DC of 30%. Note that the initial temperature was 37°C in the case of no HIFU sonication (Liang et al., 2015).

Ultrasound-mediated delivery vehicles facilitate an effective and non-invasive method for the controlled and targeted release of the drug. Recently, we have engineered an ultrasound responsive thermosensitive nanohybrid cerasomal system to enhance chemotherapy via increased intracellular accumulation of drug molecules (Liang et al., 2015). Thermosensitive nanohybrid cerasomes (HTSCs) was prepared by incorporating CFL with low temperature sensitive liposomes including 1,2-dipalmityol-sn-glycero-3-phosphocholine (DPPC), 1,2-distearoyl-snglycero-3-phosphoethanolamine-N-PEG-2000 (DSPE-PEG-2000) and 1-stearoyl-2-hydroxy-sn-glycero-3-phosphocholine (MSPC). The *in vitro* release profile demonstrated the excellent stability and longer circulation time of HTSCs, moreover release rate of DOX was sensitive to high intensity focused ultrasound (HIFU). The *in vivo* experiment depicted that cerasomes released most of the DOX within 1 min upon HIFU sonication, which significantly inhibited the tumor proliferation in a tumor-bearing mouse (Figure 3). Notably, ultrasound served as a stimulus to trigger the release of DOX from cerasomes selectively and efficiently into lesions, thus avoiding damage to healthy tissues.

In another study, pH variation has been exploited as a stimulus to control the release of DOX from cerasomal microcapsule (DCM). The DCMs exhibited excellent stability as well as biocompatibility with high drug loading content (21.86 ± 1.08%). In addition, pH-dependent sustained release of DOX greatly inhibited the growth of tumor cells *in vitro* (Zhang et al., 2014). In a natural tumor microenvironment, the redox potential between normal and tumor cells can be exploited as a stimulus for the controlled release of drugs *in vivo*. Zhou and co-workers developed a redox-responsive cerasomes through ethanol injection method. They encapsulated DOX as a model drug in nanohybrid cerasomes to evaluate the redox-dependent drug release. *In vitro* study demonstrated the higher tumoricidal activity of nanohybrid cerasomes because the lipid bilayer of vesicles could easily dissolute in the presence of glutathione (GSH) resulting in the faster release of DOX. Thus, redox-responsive cerasomes hold great potential as an intelligent drug delivery system for cancer therapy (Zhou G. et al., 2016).

#### Cerasomal NPs in phototherapeutic application

Photodynamic therapy is an external light activatable non-invasive treatment modality that involves the use of a photosensitizer (PS) which can generate cytotoxic reactive oxygen species upon exposure by a specific wavelength of light. Moreover, the non-toxic nature of applied PS and negligible dark toxicity toward normal cells endow them with an additional advantage over chemotherapy (Zhou Z. et al., 2016). However, poor water solubility and inadequate selectivity of PS have limited its therapeutic application. In order to overcome these limitations and enhance therapeutic applications, PSs can be conjugated to organic or inorganic moieties (Zhou Z. et al., 2016).

Cerasomes exhibiting highly modifiable chemical properties allow easy loading of the photoactivatable unit for effective and safe PDT. Herein, to avoid the premature release of PS from the nanocarrier, we designed and fabricated a porphyrin bilayer cerasomes (PBCs) via sol-gel reaction followed by self-assembly process from organoalkyoxysilylated lipid conjugate (PORSIL) for PDT. The resultant PBCs with an average D_hy_ of about 70 ± 13 nm showed significantly higher drug loading content (33.46%) as compared to conventional bilayer porphyrins. The blood circulation dynamics studies clearly demonstrated the improved efficacy of PBCs, measured in terms of higher tumor accumulation, better stability and enhanced biodistribution that are critical for cancer theranostics. The photodynamic studies revealed the well maintained capability of porphyrin to produce ROS even after being conjugated to cerasomes. Moreover, *in vitro* results depicted the effective tumor ablation effect of the PBCs with 400–700 nm light irradiation, revealing the potential of cerasomes as a therapeutic platform for cancer therapy (Liang et al., 2011a).

#### Multifunctional cerasomal NPs for imaging-guided therapy

**Figure 4:**
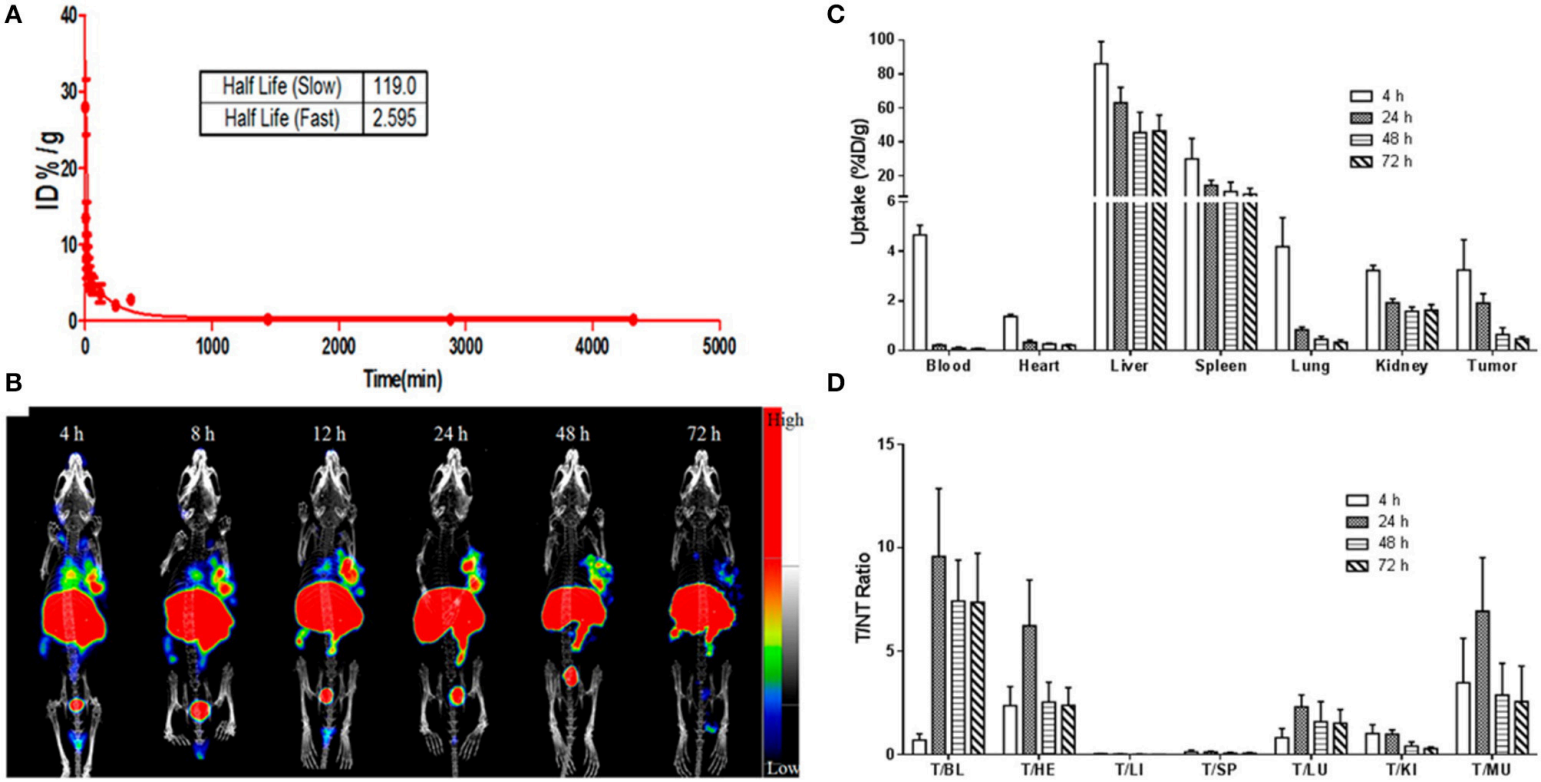
**(A)** The blood clearance curve of ICG@DPDCs-^177^Lu. **(B)** Antitumor and antimetastatic effects of PD-L1-PCI-Gd in mice with 4T1 tumors (*n* = 5) represting BLI imaging, tumor volume, and body weight. **(C)** and tumor-to-nontargeted tissue ratio **(D)** of ICG@DPDCs-^177^Lu in LLC bearing mice at 4, 24, 48, and 72 hpi (Jing et al., 2015).

Biomedical imaging techniques attributed to PET, SPECT, CT, MRI and optical imaging provide a unique tool for non-invasive and real-time tracking of therapeutic agents to the tumor site *in vivo* (Yang et al., 2015). In the recent years, there has been a remarkable level of research interest in the design and development of multifunctional nanomaterials for simultaneous imaging and therapy (Gobbo et al., 2015; Bhattarai et al., 2017). This field has been flourishing so rapidly that a new terminology “theranostics” has been coined to describe the dual functionality of such nanomaterials (Elzoghby et al., 2016; Chauhan et al., 2018). Flexible encapsulation and surface modification capability of nanohybrid cerasomes make them a promising candidate for theranostic applications. Cerasomal NPs can be further engineered to co-deliver different therapeutic and imaging agents for simultaneous monitoring of tumor cells, therapeutic agent uptake, and efficacy of treatment at the disease site. An elegant approach reported the use of a stable hybrid cerasomes for co-encapsulation of Fe_3_O_4_ NPs and an anti-cancer drug PTX (PLMCs) by thin film hydration method (Cao et al., 2014). These PLMCs demonstrated higher stability, prolonged circulation time and selective accumulation at the tumor site. Magnetic guidance-facilitated the internalization and accumulation of PLMCs into the HeLa cells thereby increasing the efficacy of PTX to inhibit tumor cells proliferation. Moreover, PLMCs exhibited much higher T_2_ relaxivity (r_2_) as compared to free Fe_3_O_4_ NPs, depicting their increased sensitivity in T_2_-weighted imaging. Such dual functional PLMCs are potentially considered to be highly important for ushering the clinical development of MR imaging-guided cancer treatment. In another recent study, our group members have revealed the ability of ^177^Lu-labeled cerasomal NPs to encapsulate indocyanine green (ICG) to provide dual-mode NIR fluorescence (NIRF) and nuclear image-guided-photothermal therapy (Jing et al., 2015). This multifunctional cerasomal NPs was synthesized from cholesterol-succinyl silane and DSPE-PEG 2000-DOTA by sol-gel reaction and self-assembly process. ^177^Lu-labeled cerasomal NPs demonstrated high tumor accumulation and good photothermal stability *in vivo* detected via NIRF and nuclear imaging. Besides, improved tumor accumulation, ^177^Lu-labeled cerasomal NPs could effectively ablate cancer cells through photothermal therapy (Jing et al., 2015; Figure 4). The ability to track cerasomal NPs is of high importance in imaging-guided-phototherapy of various tumor models.

Image-guided cancer therapy combining optical and MR imaging has attracted much attention because of their complementary capabilities for sensitive detection and detailed anatomical resolution (Biju, 2014). MRI offers a high spatial resolution, soft tissue contacts, and unlimited tissue penetration depth. On the other hand, optical imaging has higher sensitivity than MRI but has low tissue penetration depth and spatial resolution. The integration of high resolution and sensitivity into one nanocarrier significantly increases accuracy for diagnosis and subsequent therapy of cancer compared to single imaging modality (Huang et al., 2017). With the aim to improve the tumor diagnosis and treatment further, Du et al. from our lab recently fabricated a PD-L1 targeted multifunctional cerasomal NPs loaded with chemotherapeutic drug PTX and labeled with IRDye800CW and Gd-DOTA to facilitate dual mode NIRF and MR image-guided chemotherapy (Du et al., 2018). Therapeutic efficacy and targeted specificity were evaluated in mice with xenografted 4T1 breast cancer and CT26 colon tumor. Results indicated that specific and active targeting through PD-1 antibodies extended the lifetime of NIRF/MRI dual-mode therapeutic nanohybrid cerasomes (PD-L1-PCI-Gd) in the circulation. Apart from this, we also found that cerasomal NPs labeled with PD-L1 antibody initiated the recruitment of CD3^+^ and CD8^+^T cells, demonstrating excellent antitumor activity, thus satisfying the foundational principle of tumor immunology. Furthermore, PD-L1-PCI-Gd enabled non-invasive *in situ* tumor imaging with excellent sensitivity, better spatial information, high T1 relaxivity and good fluorescence imaging properties (Du et al., 2018). Such combined imaging-guided theranostic platform aimed for better spatial resolution has undoubtedly offered effective therapy via simultaneous monitoring and treatment for a plethora of diseases. However, detailed investigation regarding the long-term biodegradability, toxicity and clearance of cerasomal NPs *in vivo* would help to remove the barrier to clinical entry.

## A new generation of hybrid partially silica coated nanocarrier: bicelles

Lipid-based drug delivery vesicles have attracted considerable interest for various biomedical application, especially as drug delivery carrier because of their strong ability to self-assemble into diverse nanoformulation in an aqueous medium (Peer et al., 2007). However, despite decades of refinement in lipid base nanoformulation synthesis, the large and spherical lipid-based formulations hinder deep penetration into the core of tumor, thus limiting the antitumor efficacy. Many studies reflected that the shape of the particle could affect the therapeutic parameters including blood circulation, cellular uptake, biodistribution and pharmacokinetics (Salatin et al., 2015). It was found that disc-shaped NPs displayed preferential cellular uptake and higher microvascular adhesion than spherical NPs. Thus, lipid-based bicelles with small size and morphological versatility have emerged as a fascinating nanocarrier and are increasingly utilized in several biomedical applications (Barbosa-Barros et al., 2012).

Bicelles are usually formed by a mixture of phospholipids, e.g., 1,2- dimirystoyl-sn-glycero-3 phosphocholine (DMPC) and 1,2- dihexanoyl-sn-glycero-3-phosphocholine (DHPC) having a varying hydrophobic chain length (Lee et al., 2008). The long-chain DMPC assembles as a flat bilayer, whereas DHPC as a short-chain lipid thereby stabilizes the rim of bicelles (Claridge et al., 2013). As an emerging nanocarrier platform, bicelles exhibit integrated properties of traditional lipid-based vesicles (i.e., liposomes) and classical mixed micelles as a part of hybrid compositions (Lee et al., 2008). To date, bicelles have been successfully used to study the structure and function of membrane-bounded proteins. Additionally, bicelles equipped with a bilayer domain facilitates entrapment of various hydrophobic chemotherapeutics (Sanders and Prosser, 1998). Nevertheless, leakage of entrapped drugs due to the destabilization of bicelles after interaction with plasma proteins remains as a major challenge for its enhanced therapeutic application (Smrt et al., 2017). In order to circumvent this obstacle, Kikuchi and co-workers introduced organic-inorganic hybrid bicelles comprising of DHPC that segregated to the edge region of high curvature and long chain CFL that formed the planar surface (Yasuhara et al., 2011). Non-toxic polyorganosiloxane surface not only offers higher stability to bicelles but also facilitates conjugation of ligands easily via silane-coupler chemistry for the targeted therapy.

### Specific design strategies of organic-inorganic hybrid bicelles

Organic-inorganic hybrid bicelles are known to garner properties from their organic and inorganic components in a synergistic way. Bicelles have attracted particular attention owing to their ability to form a strong cross-linked siloxane network on their surface. This is mainly achieved by the sol-gel process followed by the poly-condensation of the organoalkoxysilane lipid resulting in final hybrid bicelles composed of silicate surface and a lipid bilayer. The first report involved in the synthesis of partially silica coated bicelles was introduced by Yasuhara et al. (2011). They prepared the hybrid bicelles by simply hydrating the mixture of long-chain organoalkoxysilane lipid and a short-chain phospholipid DHPC in a molar ratio of 7:2. The Fourier transform infrared (FT-IR) spectrum analysis revealed a significant peak of asymmetric stretching vibration of the siloxane bond (Si–O–Si, 1,100 cm^−1^) confirming a cross-linked siloxane network on the surface of hybrid bicelles. Microscopic observations further revealed an incredible morphological stability and well-defined hybrid nanodiscs with an edge-on thickness of 4.4 ± 0.4 nm. Therefore, organic-inorganic hybrid bicelles appear to be a promising candidate to increase the stability of supramolecular assembly. Besides, Kikuchi et al. also investigated the thermal stability of hybrid bicelles formed from long-chain alkoxysilane lipid and a short-chain phospholipid. Unlike conventional phospholipid bicelles with limited thermal stability, hybrid bicelles maintained their discoid morphology in water even above the phase transition temperature of long-chain forming lipids. Such remarkable structural integrity and thermal stability of bicelles could be of high importance in the field of cancer theranostics (Yasuhara et al., 2012).

### Biocompatibility aspect of hybrid bicelles

An ideal drug delivery vehicle should be inert without eliciting any intolerable toxicity, biocompatible and should retain its inherent property without altering physio-chemical attributes to perform an intended therapeutic action. Recently, our group has investigated the biocompatibility of hybrid bicelles in both cancer and immune-cell lines (Lin et al., 2016). The *in vitro* cytotoxicity analysis in HUVECs and two other immune cells, e.g., T-cells and BMDCs demonstrated negligible toxicity. It is therefore quite evident that the hybrid bicelles own good biocompatibility and can be used for a variety of therapeutic and diagnostic application both *in vitro* and *in vivo*. However, it is also important to mention that the *in vitro* screening alone necessarily doesn't address the overall biocompatibility aspects of nanocarriers *in vivo*. It's due to the fact that biological interaction of organic-inorganic hybrid is complex and discerning eventual fate of these hybrid materials *in vivo* is even more challenging. Nevertheless, it is highly recommended that the future studies on hybrid bicelles should encompass *in vivo* clearance mechanism, detail studies on biocompatibility, and most importantly the safety aspects *in vivo*.

### Effect of shape and ligand modification on cellular uptake

Although hybrid nanomaterials have gained immense attention lately, the interaction mechanism of these NPs with the biological system is the least explored. In regard to the growing interest of hybrid bicelles in the treatment and diagnosis of cancer, it is mandatory to understand the intracellular fate and cellular uptake mechanism *in vivo*. Once these nanomaterials dock on the surface of the cells, they immediately initiate dynamic nano-bio interaction between highly heterogeneous nano-bio interfaces (Kinnear et al., 2017). More intriguingly, for desirable therapeutic performance, it is particularly important to understand the relationship between the physiological properties of hybrid nanomaterial and extent of cellular uptake. It is a well-known fact that the cellular uptake of nanomaterial depends on the particle size, shape, surface charge, surface chemistry and cell type. Despite that, dosing and time-dependent administration of NPs can also influence the cellular uptake mechanism (Verma and Stellacci, 2010).

In contrast to the spherical nanomedicines, the information about cellular uptake, biodistribution, and degradation of anisotropic nanomaterial is limited. So far our group has investigated the decisive role of shape and ligand modification on the cellular uptake of hybrid bicelles (Wang et al., 2017). For this purpose, hybrid bicelles with non-modification (HB), edge modification (EHB), and plan modification (PHB) of octa-arginine sequence were synthesized from long-chain CFL and short chain DHPC phospholipid. The spherical cerasomal NPs with similar components were also fabricated for the comparative study. The data showed that shape anisotropy, functionalization anisotropy, and phagocytic/endocytic nature of cells have some correlation with cellular uptake and internalization. In order to understand the impact of shape anisotropy, the cellular internalization of HB and spherical cerasomes in four different cell lines (MM-231, MCF-7, HUVEC, RAW264.7) were studied. The data revealed that the HB nanodiscs were more favorable for endocytosis as compared to spherical cerasomes in all the tested cell-lines. This basically infers that the NPs with a larger aspect ratio (elongated NPs) are able to experience multivalent interactions with the cell, leading to the faster uptake by the cells than their spherical counterparts. Moreover, further analysis of the shape-anisotropic nanomaterials on tumor spheroids revealed stronger signals from the sample incubated with HB but not the cerasomes. This conclusion implies that the cellular uptake of nanomaterials and their penetration power into the solid tumor can be subsequently improved by the anisotropic manipulation in their shape (Wang et al., 2017).

**Figure 5:**
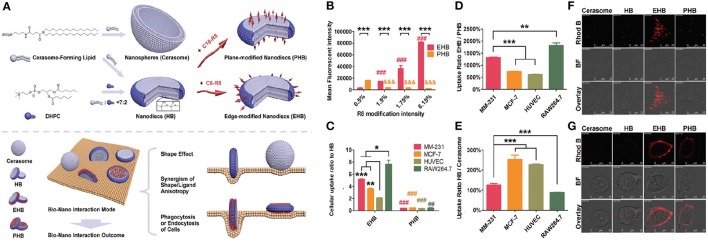
**(A)** Schematic illustration of engineering, structure, and shape of cerasome, HB, EHB, and PHB, respectively, as well as the manipulation of biological features by the anisotropy in shape and ligand modification through modulating the bio-nano interaction mode. **(B)** Cellular uptake of EHB and PHB with different R8 modification densities by flow cytometry in MM-231. Completely opposite changes on the cellular uptake were observed with the increase of R8 modification density, which was ascribed to the anisotropic modification of EHB and PHB. *n* = 3, ***p < 0.001, ###, &&&*p* < 0.001 vs. 0.5% group. **(C)** The cell uptake of EHB and PHB compared to HB on different cell lines. All the cell lines showed increased EHB internalization and decreased PHB endocytosis compared with HB, and the anisotropic modification on bicelles changed the output of their biointeraction. *n* = 3, *p < 0.05, **p < 0.01, ***p < 0.001; ##*p* < 0.01, ###*p* < 0.001 vs. EHB group. **(D)** The cell uptake of EHB compared to PHB on different cell lines. Different cell lines showed distinct EHB/PHB uptake rates, which was consistent with their different phagocytic or endocytic functions. *n* = 3, **p < 0.01, ***p < 0.001. **(E)** The cell uptake of HB compared to cerasome on different cell lines. Shape anisotropy and cell natures both affected the internalization of nanoparticulates. *n* = 3, ***p < 0.001. Cellular uptake/adhesion of Rhod B-labeled HB, EHB, and PHB by MM-231 observed using CLSM at **(F)** 37°C and **(G)** 4°C. The internalization was proved to be an energy-dependent process.

Surface modification of the hybrid bicelles is crucial for cellular uptake as it provides direct driving force (van der Waal's forces, hydrogen bonding, and electrostatic interaction) for cellular internalization (Panariti et al., 2012; He and Park, 2016). For surface modification, various ligands can be conjugated to the surface of hybrid bicelles that have a paramount influence on the interaction between nanomaterials and cells (Moros et al., 2012). These ligands have potential to significantly change the properties of the bicelles, allocating additional desirable features such as bio-derived recognition, increased circulation halftime, and weakened nonspecific interactions. The most frequently used ligands include antibodies, proteins, aptamers, peptides, cell growth factors, and other small molecules (Diller et al., 2009). Over the past few decades, significant efforts are made to understand the physical, chemical, and biological interactions of these ligands at the cell interface and subsequently their cellular uptake (Zhao J. et al., 2016). However, it is generally acknowledged that these findings cannot be generalized to all hybrid bicelles and further investigations are urgently needed. Our uptake studies in hybrid bicelles imply the cell-penetrating peptides (CPPs) of octa-arginine sequence (R8) conjugated with short alkyl chain (C8-R8) and long alkyl chain (C18-R8) resulting in the modification around the edge and plane, respectively (Wang et al., 2017). The obtained edge and plan modified hybrid bicelles were designated as EHBs and PHBs respectively. The cellular internalization of EHBs and PHBs with different R8 modified densities was studied by exposing bicelles to MM-231 cell line and uptake was quantified by flow cytometry analysis. As compared to PHBs, the increasing density of R8 ligand remarkably improved the cellular uptake of EHBs and is mainly attributed to the unique features of edge-based bio-interactions, such as larger contact angle and surface curvature at the contact site (Figure 5). In contrast, PHBs interact with a cell on its top or bottom, leading to the larger contact area, but small contact angle and surface curvature, causing less internalization of PHBs. These results are in strong agreement with already published reports concerning the effect of contact angle and surface curvature on NPs uptake (Champion and Mitragotri, 2006). For instance, particles lying parallel to the cell membrane are less likely to get internalized. In general, our findings demonstrated the importance of anisotropic CPP modification on endocytosis pathway of bicelles. Furthermore, different cell lines possess different sensitivity to EHBs and PHBs. The maximum observed difference in EHB/PHB uptake ratio was almost 12-fold between RAW264.7 and HUVECs. The observed difference in the cellular uptake of various cell types could be mainly due to the dissimilarities in the phagocytotic and endocytotic function of cells, for instance, the strong phagocytic-cells are usually more sensitive to the change in ligand location.

In addition to the modification of shape and surface of various hybrid NPs, mechanical properties such as elasticity are also hypothesized to alter the cellular uptake and tumor accumulation *in vivo*. The mechanical aspect of NPs has been exploited previously to improve the tumor accumulation due to their strong ability to bind cell receptor and squeeze through pores or passively via EPR effect. However, lack of stringent measurement strategies and reliable fabrication techniques to synthesize NPs with varying elasticity have limited their clinical validation and future application. Recently, Guo et al. reported the first experimental protocol to disseminate the underlying mechanism between elasticity and cellular uptake of NPs (Guo et al., 2018). They synthesized nanohybrid lipogels (NLGs) circumscribing an alginate core (crosslinked and uncrosslinked) and nanoliposomes (NLPs) without a hydrogel core to evaluate the effect of elasticity both *in vitro* and *in vivo*. The results demonstrated that neoplastic and non-neoplastic cells exhibited significantly greater cellular uptake of soft NLGs (Young's modulus <1.6 MPa) relative to their elastic counterparts (Young's modulus > 13.8 MPa) via two independent pathways: fusion and endocytosis, respectively. Soft NLGs depicting dominant fusion pathways could enter cells faster and at the expense of low energy compared to the hard NLGs following clathrin-mediated endocytosis. In addition, the *in vivo* tumor uptake study also demonstrated that the soft NLGs preferentially accumulated in the tumor regions compared to the higher moduli particles that accumulated especially in the liver. This study, therefore, sets a new paradigm for improving the uptake and accumulation of nanohybrid NPs at the tumor site.

### Bicelles as a potential nanocarrier for cancer theranostics

Hybrid bicelles have emerged as a versatile platform for numerous biomedical applications such as drug/gene delivery, bioimaging and cancer diagnosis (Chu et al., 2014; Lin et al., 2017b). Compared with other self-assembled NPs, namely polymersomes, micelles, liposomes and polymeric micelles, partially silica coated hybrid bicelles offers intimate advantages including good stability, high mechanical strength, controlled drug release, easy surface modification and favorable biocompatibility (Li et al., 2018). Progress in utilizing hybrid bicelles for cancer theranostics advances rapidly due to their ability to provide a robust framework in which two or more therapeutic agents can be incorporated to give multifunctional capabilities and additionally target multiple components of heterogeneous cancer cells (He et al., 2015). In the following section, we will summarize the therapeutic application of partially-silica coated hybrid bicelles in cancer treatment.

#### Chemotherapy

The ultimate goal of cancer chemotherapy is to improve the efficacy of treatment and reduce the adverse side effects of therapeutic drugs (Wang et al., 2011). Hybrid bicelles with biomembrane mimetic properties have opened a new way to increase the therapeutic index of cancer drugs by altering their tumor accumulation, pharmacokinetics, and biodistribution in a biological system (Lee et al., 2008). More importantly, the intrinsic versatility of hybrid bicelles permits incorporation of various hydrophobic drugs into their hydrophobic domains through physical entrapment (Saw et al., 2017). Additionally, drugs are usually retained within the highly stable hybrid bicelles under physiological conditions and are released upon exposure to specific stimuli such as temperature variation, treatment with the reducing agent, light irradiation and pH change (Zhong et al., 2016; Lin et al., 2017b). To date, only limited reports are available to demonstrate the application of hybrid bicelles for cancer therapy (Lin et al., 2016, 2017a,b).

Recently, Lin et al. fabricated a hybrid bicelles loaded with a hydrophobic DOX (HDOX@HBicelles) for chemotherapeutic treatment of cancer (Lin et al., 2016). Unlike liposomal cerasomes, the hybrid bicelles demonstrated excellent stability toward the surfactant solubilization, good biocompatibility, long-term storage and pH-sensitive release behavior *in vivo*. The biphasic release pattern with an increased amount of DOX corresponding to the acidic pH environment was evident from the release studies. This was mainly attributed to the unstable edge region of DHPC that triggers the drug release from the hybrid bicelles. Moreover, *in vitro* cytotoxicity studies confirmed that HDOX@HBicelles can induce similar cytotoxicity as compared to the free drug depending upon the time of incubation and concentration of bicelles. This suggests that the hybrid bicelles can be considered as a versatile and effective drug delivery platform for cancer therapy.

Surface PEGylation is known for its effective improvement in blood circulation kinetics of nanomedicines (Zhao, C. Y. et al., 2016). The incorporation of polyethylene glycol (PEG)–lipid derivatives within the bilayer of hybrid bicelles considerably prolongs their half-life by steric stabilization and reduces the RES uptake (Arranja et al., 2016; Kim et al., 2017). This has been reported in a recent study led by our group. In contrast to non-PEGylated counterparts, DOX-loaded hybrid bicelles with PEG modification showed higher cellular uptake, and adhesion, as well as high local drug accumulation than similarly sized DOX-loaded hybrid bicelles and significantly improved tumor suppression both *in vitro* and *in vivo* (Lin et al., 2017b). Thus, PEGylated hybrid bicelles with partial silica coating offer a facile and efficient strategy for improved drug delivery and enhanced chemotherapy.

#### Image-guided combination therapy

**Figure 6:**
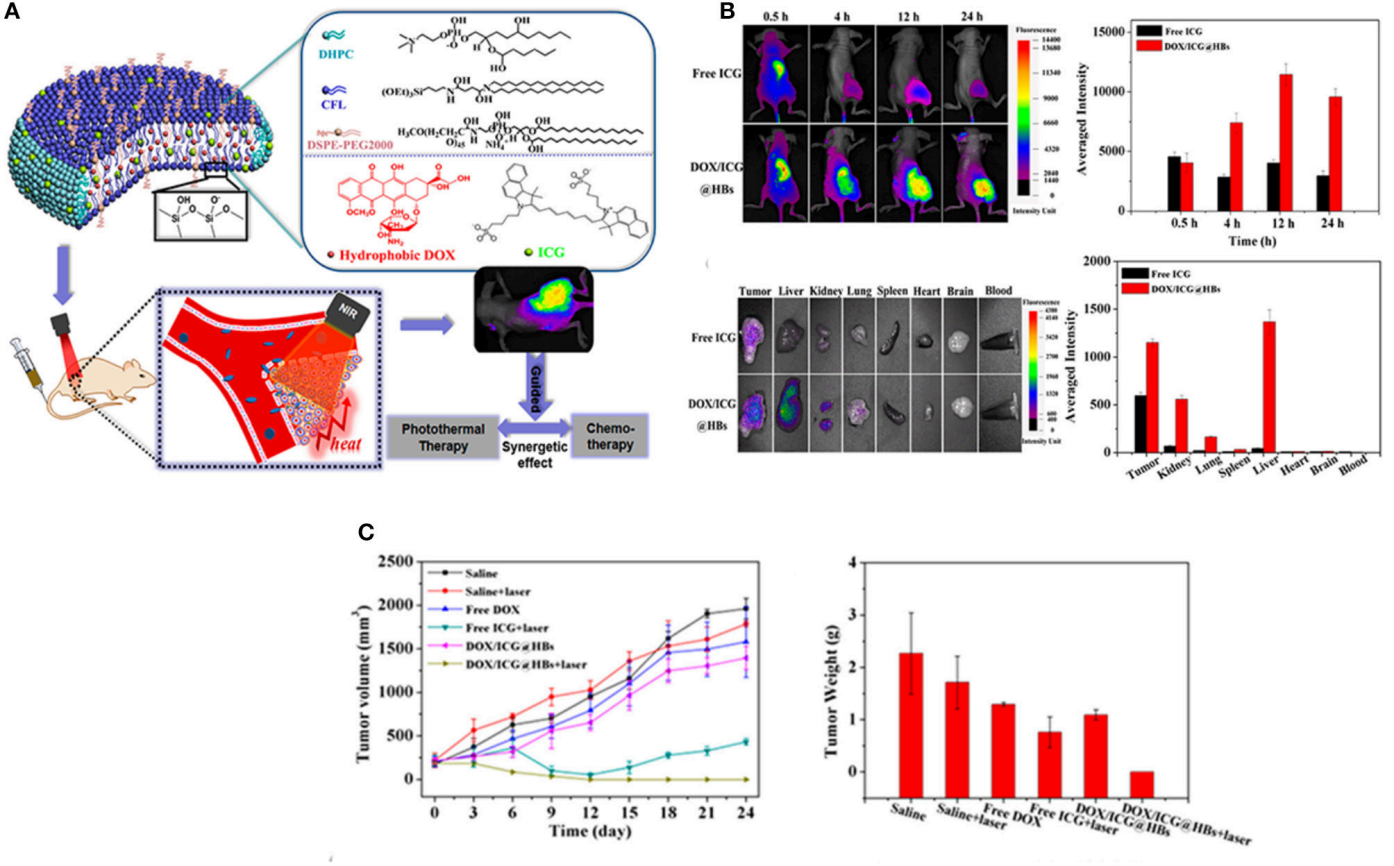
**(A)** Schematic representation of DOX/ICG@HBs for fluorescence imaging guided chemo/photothermal therapy of cancer. **(B)** *In vivo* fluorescence imaging and biodistribution of MDA-MB-231 tumor-bearing BALB/c nude mice after intravenous injection of free ICG and DOX/ICG@HBs. **(C)** *In vivo* chemo/photothermal therapy and biocompatibility of DOX/ICG@HBs on 4T1 tumor-bearing BALB/c mice upon the 808 nm laser irradiation (2 W/cm^2^, 10 min) (Lin et al., 2017a).

Although chemotherapy is widely adopted in clinical settings, it still encounters various drawbacks, such as low efficacies, multidrug resistance, tumor metastasis and severe adverse reactions (Du et al., 2017). Consequently, greater efforts have been devoted exploring alternative route of effective therapies, such as photothermal and photodynamic therapy that can enhance the concurrent limitations of chemotherapy (Peng et al., 2011). Among these, the combination therapy, particularly chemo/photothermal therapy have received considerable attention not only for fundamental research but also at clinics (Hu et al., 2017). Along with other nanomedicines, hybrid bicelles can be exploited to deliver therapeutic drug and photothermal agent simultaneously to malignant cells (Mouli et al., 2013). To this end, Lin et al. from our group recently synthesized multifunctional hybrid bicelles (DOX/ICG@HBs) co-loaded with hydrophobic DOX and ICG for NIRF imaging-guided chemo/photothermal therapy of cancer (Lin et al., 2017a). Such multifunctional bicelles demonstrated outstanding tumor targeting ability for deep tumor penetration, selective drug release behavior and subsequently facilitated fluorescence imaging guided photothermal therapy, all in one platform. DOX/ICG@HBs with an average D_hy_ of 60 nm released more than 85% of cargo at a higher temperature (50°C) and demonstrated superior chemo-photothermal cytotoxicity to MDA-MB-231 cancer cells under laser irradiation both *in vitro* and *in vivo*. It is therefore quite apparent that DOX/ICG@HBs in combination with NIR laser irradiation holds great potential for efficient cancer therapy without producing any appreciable off-target toxicity (Figure 6). Despite the paramount research progress in NPs based combination therapy, the potential application of DOX/ICG@HBs is still in its infancy, thus, further investigations are warranted to explore the additional use of such hybrid bicelles in cancer theranostics.

## Conclusion: limitations and future directions

∂SiNHs, cerasomes, and bicelles, are one of the emerging cancer treatment modalities that have strong capability to inhibit tumor growth. The key advantages of this hybrid system, such as: (a) tuneable size and shape, (b) excellent biocompatibility, (c) higher physiological stability, and (d) topologically amenable organic-inorganic domains that can be easily functionalized, make them well suited for safe and efficient conveyance of a wide variety of theranostic agents. Furthermore, for better therapeutic outcomes, it is important to customize the ∂SiNHs to selectively target the diseased tissues of the body and liberate toxic-dose of therapeutic moieties in a controlled manner over time. It can only be achieved by using ligand modified versatile nanohybrids that have the ability to (i) recognize specific target on cancer cells (ii) substantially improve the delivery of therapeutic agents to tumor region, and (iii) selectively guide within the compartments of tumor cells (e.g., the nucleus or the lysosomes). Decorating ∂SiNHs with ligands, such as CPPs, are already proven as an effective strategy to enhance the cellular uptake and accumulation in the targeted tumor cells. Last decade has witnessed the development of sophisticated ligand modified multifunctional ∂SiNHs that can be expanded further to control the spatial and temporal release of entrapped cargos against special cues such as internal or external stimuli. These multifunctional and smart ∂SiNHs can be revisited to overcome the major oncological challenges, including metastasis, tumor hypoxia, and recurrence, with the aim to achieve maximum therapeutic benefit.

Although no any official evidence is currently available for the complete clinical safety assessment and toxicity evaluation of self-assembled ∂SiNHs, researchers have already started a series of preliminary studies to evaluate their potential toxicity. As compared to conventional inorganic nanocarriers such as mesoporous silica NPs, the use of clinically approved organic lipids and facile synthesis mechanism, e.g., self-assembling technique that eliminates the need of any toxic solvents substantially ensures the clinical safety of ∂SiNHs.

In a downside, these nanohybrids (∂SiNHs) are inevitable from several physical and biological limitations, nevertheless, it is quite possible that the future works aimed to surmount these shortcomings can obviously solve the present unmet clinical needs in cancer therapy. As compared to free drugs, nanohybrids that are properly engineered and optimized have the inherent capability to enhance the accumulation of therapeutic/diagnostic agent into the tumor site. For instance, it is critical to ensure the perfect coordination between the organic-inorganic domains of nanohybrids and loaded therapeutic agents. Moreover, with multiple functions and modification, the architecture of ∂SiNHs become quite complex which can lead to the difficulties in their robust and reproducible manufacturing process. Incorporation of multiple drugs, imaging agents, and ligands within a nanohybrid on a large scale might be expensive and challenging as well. That's why further studies and optimizations are still required to eventually scale up the manufacturing process of designed nanohybrids from laboratory research to clinical application. Other limitations centered on detailed cellular uptake mechanism, clearance/degradation pathways after the intended action, and toxicological evaluation/ safety profile of these hybrid carriers both *in vitro* and *in vivo* are yet to be resolved. This instigates the need for further systematic investigations to explore the safety issues, pharmacokinetics and biodistribution parameters to fulfill the standard clinical requirements.

It is a broadly established fact that, despite the potential that inorganic component (Si) of ∂SiNHs have offered for oncological application, their clinical translation are still challenging. It is highly desirable that these nanohybrids could be easily removed from the body via renal clearance mechanism following a natural degradation process *in vivo*. Hence, a better understanding of biodegradation and renal clearance mechanism in future would help to develop and modify the nanohybrids to achieve better therapeutic outcomes. In a recent study, Song et al. designed a nanohybrid system for MRI-guided photothermal therapy via the self-assembly of photothermal dye and renal clearable ultra-small iron oxide NPs (Song X. et al., 2014). Similarly, Sheng et al. investigated the dendritic degradable mesoporous SiNPs through a simple biphase stratification approach (Shen et al., 2014). Such studies provide substantial evidence to improve the clinical efficiency of ∂SiNHs by designing nanomaterials with comparable efficacy and reduced long-term health risks in future. Nevertheless, ∂SiNHs show high potential for therapeutic applications, especially in oncology. We are optimistic that these hurdles could eventually be overcome by effective participation and collaborations between chemists, biologists, material scientists, and clinicians.

## Author contributions

SH wrote and revised the manuscript. PB revised the manuscript. ZD conceived the idea of the paper, revised the manuscript throughly and supervised the whole writing process.

### Conflict of interest statement

The authors declare that the research was conducted in the absence of any commercial or financial relationships that could be construed as a potential conflict of interest.
